# Successful Enucleation of Fibroid, after Laparotomy

**Published:** 1887-06

**Authors:** John S. Dickson

**Affiliations:** Pittsburgh, Pa.


### CASE OF SUCCESSFUL ENUCLEATION, AFTER LAPAROTOMY, OF A FIBROID ATTACHED TO THE FUNDUS UTERI. BY JOHN S. DICKSON, M. D., PITTSBURGH, PA.

Mrs. R.——, Pa., æt., 24, married, had given birth to two stillborn children, one at full term and the other at an earlier period of pregnancy. She was sent to me from near Cornellsville (by Dr. Phillips of that place) September 1, 1886, and was taken to Mercy Hospital after being examined by Dr. James McCann and myself at my office.

*History*—For three years patient had been suffering from an abdominal tumor, which, during the last few months previous to her reporting to me, had increased rapidly in size.

The diagnostic features indicated a fibroid growth attached to the fundus of the womb on the right side. The os uteri was pulled up on the left side to an extent which made it impossible to use a sound or make a digital examination.

The patient rested quietly at the hospital until Tuesday, September 7, when laparotomy was performed. The incision was made about 13 inches in length, extending nearly from the symphysis pubis to the ensiform cartilage. The tumor was found to be a fibroid of a firm, elastic consistency, and intimately adherent to the fundus uteri on the right side. At first it seemed impossible to remove the tumor without the uterus, but eventually it was decided to enucleate the growth from its attachments. The peritoneal sac, from which the tumor was enucleated, was very large, and the womb itself was about four times its normal size. This, at first, gave the impression that the growth was intra-uterine. The enlargement was found to he symmetrical and regular in form, however, and was then attributed to inflammation. It was considered probable, that, after removal of the tumor, the womb would atrophy to its natural dimensions, and hysterectomy was therefore rejected. During the operation a great many ligatures (over forty) were required to control hemorrhage.

After the growth had been removed, the edges of the rent in the peritoneal covering or sac were approximated by sutures, and the whole let fall back into the abdomen. The abdominal wound was brought together by interrupted sutures of silver wire, eleven in number. A rubber drainage-tube was inserted between the two last sutures, dipping down into the sac. The wound was then dressed with lint, moistened in bi-chloride solution, and linen gauze placed over that. Scarcely any blood was lost during the operation, and the patient evinced no evidence of shock at any time. The tumor was weighed, and found to be eleven pounds, two ounces in weight. At the operation Drs. James McCann, Richardson, and Davis and the hospital staff assisted.

Patient showed but little disturbance from the anæsthetic, but complained of pain some two or three hours after the operation.

She was relieved by morphia. She had considerable pain, and was restless during first night.

Twelfth day: Patient taken out of bed, and put in a large chair for an hour. On each subsequent day she sat up for a little longer period. She is becoming less restless, and eats and sleeps well. Bowels move naturally.

During the period of eleven days intervening between the operation and the day on which she left her bed for the first time, the patients diet consisted entirely of J. P. Bush Manufacturing Company's Bovinine, with a little milk and stimulants. Other foods were tried, but she could not retain them.

On the 19th day the patient was discharged from the hospital.

[Record of pulse omitted.]

October 20, 1886, patient had a miscarriage, at her parent's home, in Mt. Pleasant, Pa., and the fœtus was supposed to have been of four months' development at the time of the abortion. The peculiarities in the size and shape of the uterus, which were considered at the time of the operation to be due to inflammation, were thus explained.

November 11, 1886, patient came to my office to show how well she was. She still had, occasionally, a little discharge from the sinus, where the drainage-tube had been inserted. Extending down towards the right inguinal region, from the sinus, was a noticeably thickened and indurated condition of the parts. This was considered the source of the discharge, and tenderness was noticeable here when the flow was interrupted for any length of time. The induration, however, according to the patient's statement, was gradually decreasing. Patient was otherwise stout and in good health.

It will be conceded that the above record of the case is a remarkable one as to results. The operation occupied 51 minutes, and necessarily subjected the patient to a severe strain. It certainly is remarkable that at no time the patient's pulse was higher than 96 nor her temperature higher than 101°. Many of the good results I attribute to the use of bovinine, which was practically the patient's only nutriment, during the first eleven days, and the careful employment, during the entire case, of antiseptic precautions.—*Gaillard's Journal for May.*

Page(s) missing

